# Silencing DSCAM-AS1 suppresses the growth and invasion of ER-positive breast cancer cells by downregulating both DCTPP1 and QPRT

**DOI:** 10.18632/aging.103538

**Published:** 2020-07-27

**Authors:** Zhang Yue, Jia Shusheng, Song Hongtao, Zhao Shu, Huang Lan, Zhang Qingyuan, Cheng Shaoqiang, Huang Yuanxi

**Affiliations:** 1 Department of Medical Oncology, Harbin Medical University Cancer Hospital, Harbin, China; 2 Department of Breast Surgery, Harbin Medical University Cancer Hospital, Harbin, China; 3 Department of Pathology, Harbin Medical University Cancer Hospital, Harbin, China

**Keywords:** DSCAM-AS1, DCTPP1, QPRT, miRNA, H3K27 acetylation

## Abstract

Breast cancer (BC) remains a significant threat to the health of women; however, the mechanism underlying the initiation and progression of BC is poorly understood. We analyzed data from the Gene Expression Omnibus database and The Cancer Genome Atlas datasets to identify differentially expressed genes between BC and normal tissues. The roles of dCTP pyrophosphatase 1 (DCTPP1) and quinolinate phosphoribosyltransferase (QPRT) in BC cells were investigated after knocking down or overexpressing the genes. The regulatory effects of Down syndrome cell adhesion molecule antisense RNA 1 (DSCAM-AS1) on DCTPP1 and QPRT expression were determined using luciferase reporter, RNA immunoprecipitation, RNA pull-down, chromatin immunoprecipitation, and fluorescence *in situ* hybridization assays. DCTPP1 and QPRT were overexpressed in BC compared to normal tissues. Overexpression of DCTPP1 and QPRT was associated with poor BC progression and promoted growth, migration, and invasion of MCF7 and T47D cells but inhibited apoptosis. DSCAM-AS1 increased QPRT expression via competitively binding miRNA-150-5p and miRNA-2467-3p. DSCAM-AS1 promoted *DCTPP1* gene transcription by affecting H3K27 acetylation and enhanced *DCTPP1* mRNA stability by binding to the 3′ untranslated region, which collectively resulted in DCTPP1 overexpression. Overall, DSCAM-AS1 knockdown decreased both DCTPP1 and QPRT expression, inhibiting the growth, migration, and invasion of estrogen receptor-positive BC.

## INTRODUCTION

Breast cancer (BC) is one of the most common malignant cancers affecting females. Global cancer statistics (GLOBOCAN) 2018 predicted that approximately 2.1 million new BC cases would occur in the year 2019 worldwide [1]. Estrogen receptor (ER)-positive BC constitutes the main disease subtype. Because ER-positive BC is initially responsive to neoadjuvant endocrine therapy, patients with ER-positive BC generally have a better prognosis than patients with ER-negative BC. However, most BC deaths occur in women with ER-positive BCs, as the incidence of ER-positive disease is much higher than that of ER-negative BC (approximately 75% compared to 25%) [2–4]. Moreover, a substantial fraction of patients either have inherent or acquired endocrine therapy resistance [2–4]. However, the mechanism underlying the initiation and progression of BC is poorly understood, highlighting the urgent need to understand the pathological mechanisms.

Human dCTP pyrophosphatase 1 (DCTPP1), which belongs to the nucleoside triphosphate pyrophosphatase (NTP-PPase) superfamily, plays an important role in maintaining genomic fidelity, stability, and integrity [5]. Many pathophysiological processes, such as cell metabolism, oxidative damage, and pathogen infection tend to generate noncanonical nucleotides like dUTP, 2-hydroxy-dATP, and 8-oxo-dGTP. Incorporation of noncanonical nucleotides into DNA likely leads to DNA mutagenesis and damage, resulting in decreased cell proliferation and even cell death. DCTPP1 hydrolyses the α–β phosphodiester bond of deoxyribonucleotide triphosphates (dNTPs) to produce the corresponding monophosphate and inorganic pyrophosphate (PPi), which can avoid the incorporation of noncanonical nucleotides into mitotic DNA [6]. Many scholars believe that the abnormal vigorous growth and metabolism of cancer cells requires a large amount of nucleotide triphosphate (NTP) to ensure DNA replication accuracy [6–8]. DCTPP1 is present in both the cytosol and nucleus, and accumulates in the nucleus of multiple carcinomas, including lung, breast, liver, cervical, gastric, and esophagus cancer compared to paired adjacent tissues [6]. Additionally, treatment of BC MCF-7 cells with hydrogen peroxide (H_2_O_2_) also causes the nuclear accumulation of DCTPP1, suggesting a DNA protective effect [6].

Quinolinate phosphoribosyltransferase (QPRT) catalyzes the transfer of tryptophan to produce nicotinic acid mononucleotide, a precursor of *de novo* biosynthesis of the crucial coenzyme nicotinamide adenine dinucleotide (NAD) [9]. NAD exists in oxidized (NAD+, electron acceptor) and reduced (NADH, electron donor) forms. NADH is essential for oxidative phosphorylation in the cellular respiratory chain and is associated with multiple processes, including genomic repair and stability, chromatin modulation, calcium homeostasis, apoptosis, and aging. Many cancer cell types rely on a large amount of NADH to sustain their rapid growth and maintain DNA integrity after exposure to DNA toxic agents [9, 10]. Targeting of enzymes like tryptophan-2, 3-dioxygenase, and nicotinamide phosphoribosyltransferase in the *de novo* NAD synthesis pathway triggers or sensitizes cancer cells to apoptosis [11–13]. Thus, pharmaceutical inhibitors of these enzymes have been developed and they are now being used in clinical trials [11–13]. Nevertheless, the role of QPRT in BC has seldom been investigated.

Long noncoding RNAs (lncRNAs) have recently been implicated in a variety of tumor processes, including carcinogenesis, tumor growth, and drug resistance [14–16]. lncRNAs play an important role in regulating gene expression at both pre- and post-transcriptional levels. Aberrant lncRNA expression causes abnormal expression of genes, many of which are involved in carcinogenesis [14–16]. One well-studied molecular function of lncRNAs in gene expression regulation is their competing endogenous RNA (ceRNA) activity. lncRNAs can competitively bind miRNA, which decreases the abundance of miRNA binding to the 3' untranslated region (UTR) of mRNA and thus attenuates miRNA-mediated degradation of target mRNA [17]. Additionally, several molecular functions of lncRNAs in gene regulation have been uncovered. lncRNAs can function as “scaffolds,” “decoys,” signal transducers, and regulatory factors in *cis* or *trans* to enhance or inhibit gene expression [18, 19]. For example, the lncRNA P21-associated ncRNA DNA damage-activated (PANDA) has been found to act as a “decoy” of the nuclear transcription factor Y subunit alpha (NF-YA) [20]. The binding of NF-YA to lncRNA PANDA limits NF-YA binding to the promoter of many pro-apoptotic genes, resulting in rapid osteosarcoma cell proliferation. DSCAM-AS1 has been identified from 58,648 lncRNAs in 947 BC samples by Niknafs et al., owing to its critical role in BC biology and drug resistance, but the underlying mechanism has not been fully elucidated [4]. DSCAM-AS1 localizes to both the cytoplasm and the nucleus, suggesting complex regulatory effects. DSCAM-AS1 was strongly induced by the ER, which was confirmed as an important transcription factor initializing DSCAM-AS1 expression. High DSCAM-AS1 expression conversely confers BC resistance to tamoxifen, an inhibitor of the ER [4].

The NCBI Gene Expression Omnibus (GEO) and The Cancer Genome Atlas (TCGA) databases include data relative to gene expression profiles in many cancer cells and tissues. We analyzed the data and found that DCTPP1 and QPRT were overexpressed in BC tissue compared to normal tissue. Highly expressed DCTPP1 and QPRT were associated with poor BC progression. Therefore, this study investigated the effects of DCTPP1 and QPRT on the growth, apoptosis, migration, and invasion of BC cells. Bioinformatics analysis further revealed that expression of both DCTPP1 and QPRT was positively regulated by the lncRNA DSCAM-AS1 via divergent mechanisms. This study demonstrated that DSCAM-AS1 silencing suppressed the growth and invasion of ER-positive BC cells by downregulating both DCTPP1 and QPRT.

## RESULTS

### DCTPP1 and QPRT were highly expressed in BC tissues compared to normal tissues

**Figure 1:**
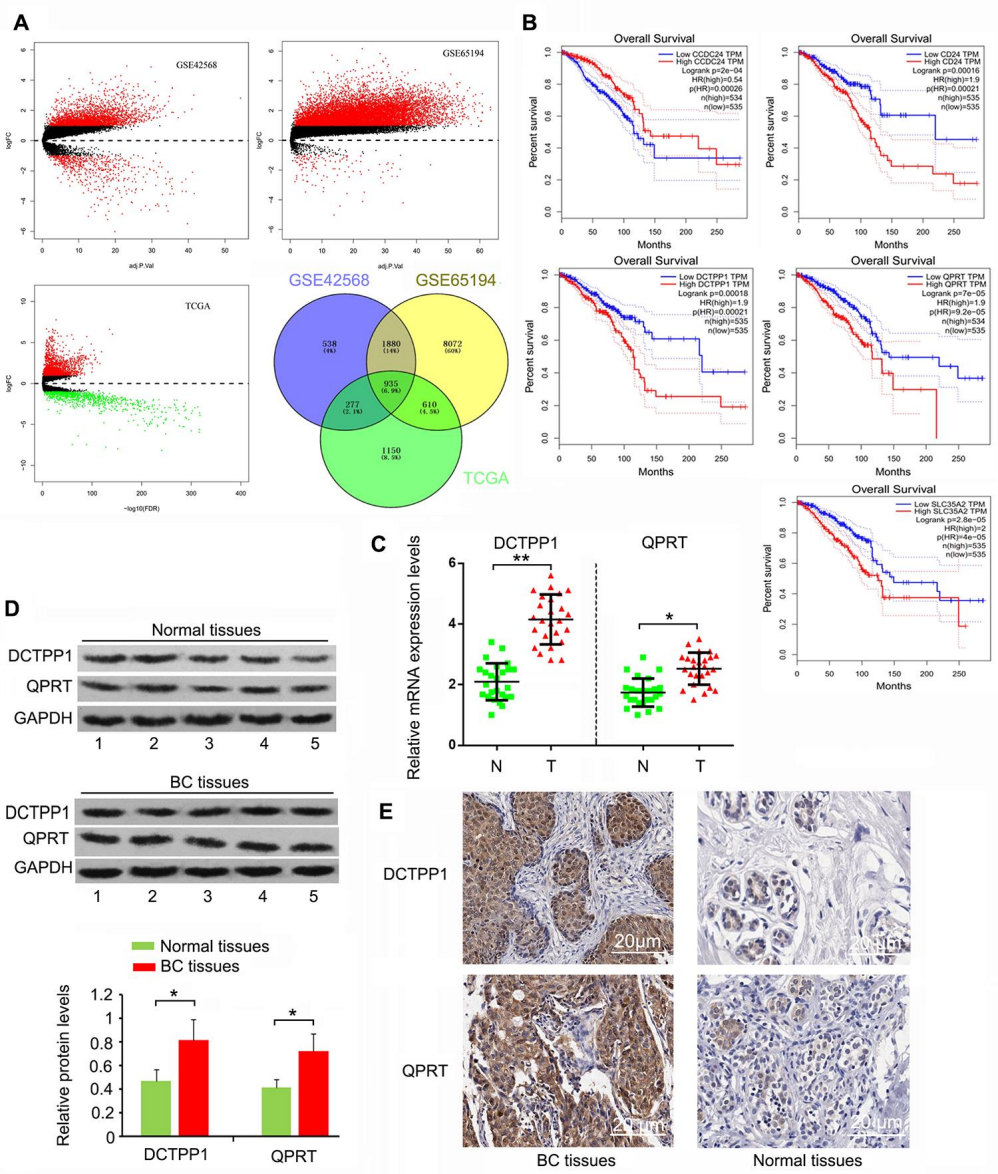
**Highly expressed** DCTPP1 and **QPRT in BC tissues compared to normal tissues.** (**A**) A total of 935 genes, whose expression was different between BC and normal tissues, were screened from GSE42568, GSE65194, and TCGA expression datasets. (**B**) The expression correlation of these genes to the prognosis of patients with BC was analyzed using GEPIA web (http://gepia.cancer-pku.cn/). We found five protein-coding genes, including *CCDC24, CD24, DCTPP1, QPRT*, and *SLC35A2*, whose expression was correlated with BC prognosis. DCTPP1 and QPRT expression levels in BC and normal breast tissues were tested using PCR (**C**), western blot (**D**) and IHC assays (**E**). DCTPP1 and QPRT expression levels were confirmed to be higher in BC tissues than in normal breast tissues. *P < 0.05 and **P < 0.01 vs. normal breast tissues. N: normal breast tissues; T: breast tumor tissues.

We initially screened 935 genes that showed notable upregulation or downregulation between BC and normal tissues in the GEO GSE42568, GSE65194, and TCGA expression datasets (Figure 1A), and then analyzed the expression correlation of these genes to the prognosis of patients with BC using Gene Expression Profiling Interactive Analysis (http://gepia.cancer-pku.cn/). The top five protein-coding genes whose expression was positively and most closely correlated to poor BC prognosis were *SLC35A2*, *QPRT*, *CD24*, *DCTPP1*, and *CCDC24* (Figure 1B). To date, the roles of *CCDC24* and *SLC35A2* have not been analyzed in cancer-related studies. In contrast, CD24 has been systematically investigated in various cancers. QPRT and DCTPP1 have been reported, but only in a few types of cancers. This study hence focused on the roles of QPRT and DCTPP1 in BC. This study collected 27 BC samples. Cancer staging was determined as stage 1 to 4 according to the Union for International Cancer Control TNM classification system [21]. Pathological grading (G1-G3) was performed according to the modified Scarff-Bloom-Richardson grading system [22]. As indicated by polymerase chain reaction (PCR) and western blotting, DCTPP1 and QPRT mRNA and protein levels were higher in BC than in normal tissues (*P* < 0.05 or *P* < 0.01, Figure 1C and D). According to immunohistochemistry (IHC) results, DCTPP1 localized to both the cytoplasm and nucleus of breast cells, but primarily accumulated in the nucleus (Figure 1E). This phenomenon was also observed in previous studies. QPRT only accumulated in the cytoplasm, and the enrichment of QPRT in BC tissues was higher than in normal breast tissue. These results suggested that DCTPP1 and QPRT had important roles in BC development.

### DCTPP1 and QPRT promoted BC cell growth and invasion

**Figure 2:**
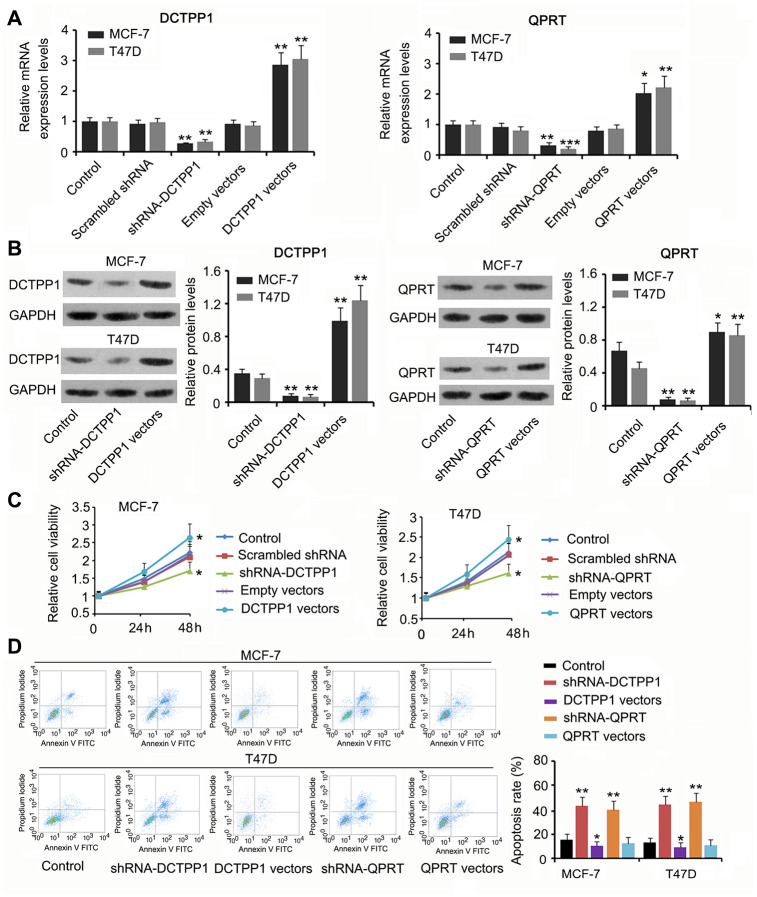
**The regulatory effects of** DCTPP1 and **QPRT on BC cell growth and apoptosis.** DCTPP1 and QPRT mRNA (**A**) and protein (**B**) levels in MCF-7 and T47D cells were changed after transfection with shRNA-DCTPP1, shRNA-QPRT, and DCTPP1 and QPRT expression vectors. (**C**) Down-regulation of DCTPP1 or QPRT was associated with reduced viability in both MCF-7 and T47D cells and increased DCTPP1 or QPRT was associated with increased cell viability. (**D**) The apoptosis rate of MCF-7 and T47D cells increased after knocking down either DCTPP1 or QPRT. DCTPP1 overexpression decreased the apoptosis rate of MCF-7 and T47D cells, and QPRT overexpression only marginally decreased MCF-7 and T47D apoptosis. *P<0.05, **P<0.01 and ***P<0.001 vs. control group.

We suppressed or promoted DCTPP1 and QPRT expression in BC cells to explore their effect on cell growth and invasion. DCTPP1 mRNA and protein levels in MCF-7 and T47D cells decreased 48 h after transfection with short hairpin RNA (shRNA)-DCTPP1 (*P* < 0.01, Figure 2A and 2B) and increased after transfection with the expression vectors (*P* < 0.01). Transfection with shRNA-QPRT caused downregulation of QPRT in MCF-7 and T47D cells (*P* < 0.01 or *P* < 0.001). Transfection with the expression vector conversely increased QPRT mRNA and protein levels (*P* < 0.05 or *P* < 0.01). Downregulation of DCTPP1 or QPRT was associated with reduced viability in both MCF-7 and T47D cells (*P* < 0.05, Figure 2C), and increased DCTPP1 or QPRT expression was associated with increased cell viability (*P* < 0.05). The apoptosis rate of MCF-7 and T47D cells increased after knocking down either DCTPP1 or QPRT (*P* < 0.01). DCTPP1 overexpression decreased the apoptosis rate of MCF-7 and T47D cells, and QPRT overexpression only marginally decreased MCF-7 and T47D apoptosis.

**Figure 3:**
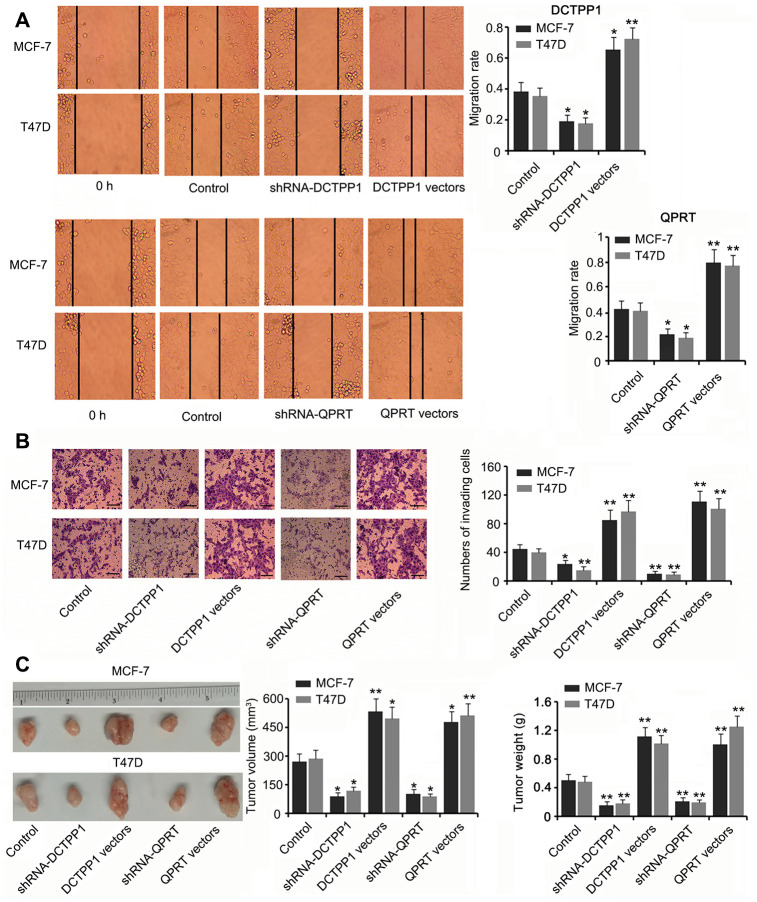
****DCTPP1 and **QPRT promoted the invasion and growth of BC cells.** MCF-7 and T47D cells were transfected with shRNA-DCTPP1, shRNA-QPRT, and DCTPP1 and QPRT expression vectors. The migration (**A**) and invasion (**B**) of BC cells were inhibited after DCTPP1 and QPRT knockdown, but enhanced after DCTPP1 and QPRT overexpression. The bar in the pictures (**B**) indicates a length of 5 μm. (**C**) As indicated by the tumour xenograft assay, silencing DCTPP1 or QPRT inhibited the growth of MCF-7 and T47D cells in nude mice. MCF-7 and T47D cells overexpressing DCTPP1 or QPRT showed notably faster growth than that by MCF-7 and T47D control cells. *P<0.05 and **P<0.01 vs. control group.

DCTPP1 and QPRT knockdown inhibited the migration and invasion of BC cells (*P* < 0.05 or *P* < 0.01, Figure 3A and B) while DCTPP1 and QPRT overexpression enhanced the migration and invasion of BC cells (*P* < 0.05 or *P* < 0.01). A tumor xenograft assay demonstrated the tumor-promoting effects of the *DCTPP1* and *QPRT* genes. Silencing DCTPP1 or QPRT inhibited the growth of MCF-7 and T47D cells in nude mice (*P* < 0.05, Figure 3C). MCF-7 and T47D cells overexpressing DCTPP1 or QPRT showed notably faster growth than did MCF-7 and T47D control cells (*P* < 0.05 or *P* < 0.01).

### DSCAM-AS1 was involved in DCTPP1 and QPRT expression in ER-positive BC cells

**Figure 4:**
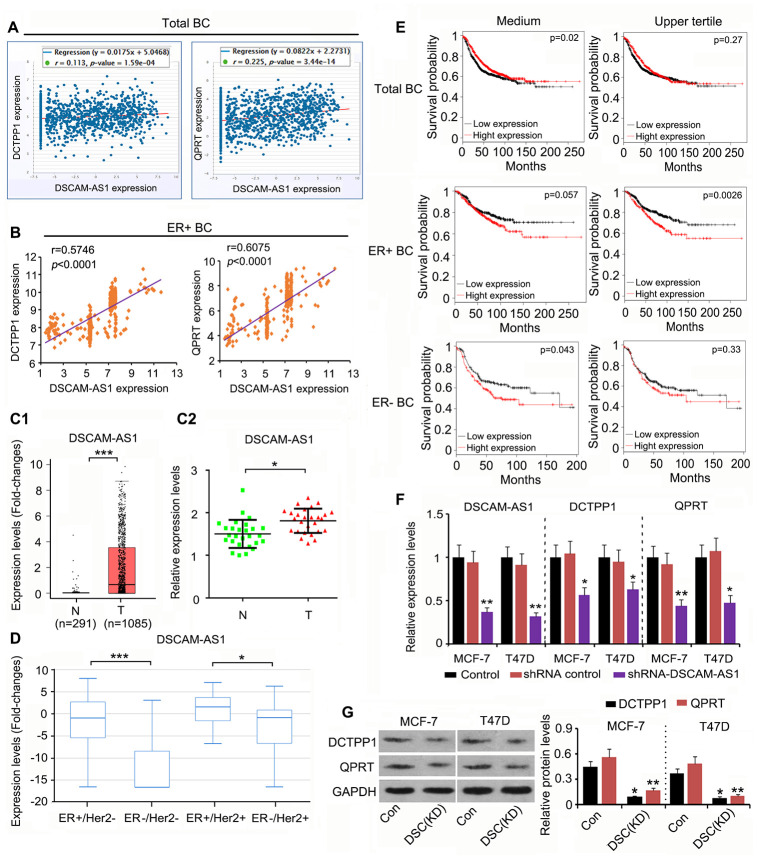
**DSCAM-AS1 positively regulates** DCTPP1 and **QPRT expression in ER-positive BC cells.** (**A**) According to Starbase web (https://web.archive.org/web/20110201054358/http://starbase.sysu.edu.cn/clipSeq.php), DSCAM-AS1 expression was positively correlated with both DCTPP1 (*P*=1.59×10^-4^) and QPRT (*P*=3.44×10^-14^) expression in BC. (**B**) Remarkably, the correlation coefficient is higher in ER-positive BC (data from GSE6532 dataset) than in the total BC. DSCAM-AS1 was also up-regulated in BC tissues according to the TCGA database (**C1**) and our PCR results (**C2**). (**D**) Data in TCGA database further showed that DSCAM-AS1 expression was higher in ER-positive BC than that in ER-negative BC. (**E**) As indicated by TCGA database, higher DSCAM-AS1 expression was associated with better prognosis in patients with BC. However, up-regulated DSCAM-AS1 was conversely associated with a poor prognosis in both ER-positive and ER-negative BC patients. Notably, high expression of DSCAM-AS1 was correlated with very poor prognosis when BC-positive patients were divided by the upper quartile value of the DSCAM-AS1 expression, and not the medium value. (**F**) PCR assay was performed to detect DSCAM-AS1, DCTPP1 and QPRT expression after DSCAM-AS1 knockdown. (**G**) Western blot was performed to detect DCTPP1 and QPRT protein levels after DSCAM-AS1 knockdown. B1-B2: *P<0.05 and ***P<0.001 vs. Normal breast tissue group. (**E**, **F**) *P<0.05 and **P<0.01 vs. control group. DSC(KD): DSCAM-AS1 knockdown.

Although DCTPP1 and QPRT upregulation promoted the growth and invasion of BC cells, the mechanism responsible remained unclear. This study analyzed the expression of genes that were correlated with DCTPP1 and QPRT expression using the data in the GSE42568 and Starbase datasets (https://web.archive.org/web/20110201054358/http://starbase.sysu.edu.cn/clipSeq.php). DSCAM-AS1 expression was positively correlated with both DCTPP1 (r = 0.113, *P* = 1.59×10^-4^) and QPRT (r = 0.225, *P* = 3.44×10^-14^) expression in BC (Figure 4A). Remarkably, the correlation coefficient was higher in ER-positive BC (data from GSE6532 dataset) than in the total BC data (Figure 4B). DSCAM-AS1 was also upregulated in BC tissues according to TCGA database (*P* < 0.001, Figure 4C1) and our qPCR results (*P* < 0.05, Figure 4C2). Data from TCGA database further showed that DSCAM-AS1 expression was higher in ER-positive BC than in ER-negative BC (Figure 4D). These data agreed with a previous report that DSCAM-AS1 is transcriptionally regulated by the ER. A seemingly paradoxical result provided by TCGA database was that higher DSCAM-AS1 expression was associated with better prognosis in patients with BC (Figure 4E). However, upregulated DSCAM-AS1 was conversely associated with a poor prognosis in both ER-positive and ER-negative BC patients. Notably, high expression of DSCAM-AS1 was correlated with a very poor prognosis when BC-positive patients were divided by the upper quartile value of the DSCAM-AS1 expression, not the median value (*P* = 0.0026). According to a previous study, DSCAM-AS1 is highly expressed in ER-positive BC cell lines, such as MCF-7 and T47D. To determine whether DCTPP1 and QPRT expression was regulated by DSCAM-AS1 in ER-positive BC cells, we knocked DSCAM-AS1 down in two ER-positive BC cell lines. Transfection with shRNA-DSCAM-AS1 decreased DSCAM-AS1 expression in MCF-7 and T47D cells (*P* < 0.01, Figure 4F), which resulted in decreased DCTPP1 and QPRT mRNA and protein levels (*P* < 0.05 or *P* < 0.01) in ER-positive BC cells (Figure 4F and 4H).

### DSCAM-AS1 regulated QPRT expression by competitively binding miRNAs

**Figure 5:**
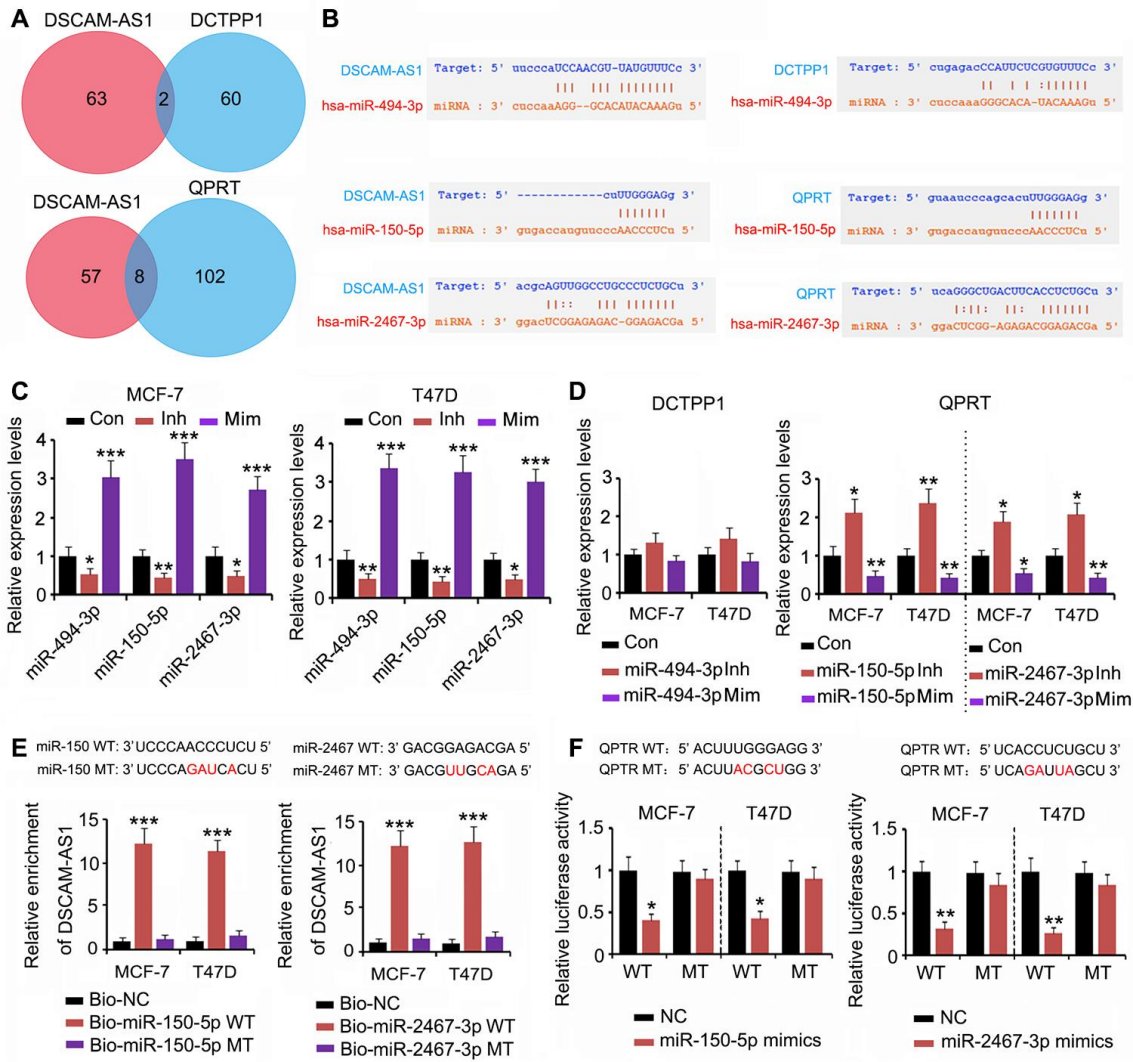
**DSCAM-AS1 regulated QPRT expression by sponging miRNAs.** (**A**) miRNAs targeting DSCAM-AS1, DCTPP1 and QPRT were predicated using PITA, RNA22, miRmap, mircoT, miRanda, PicTar, and Targetscan softwares. Venn diagram was used to find miRNAs targeting both DSCAM-AS1 and DCTPP1 or QPRT. (**B**) miRNA-494-3p was predicted to tightly bind to DSCAM-AS1; however, the connection between miRNA-494-3p and the *DCTPP1* mRNA seem to be weak. miRNA-150-5p and miRNA-2467-3p were predicted to tightly bind to both DSCAM-AS1 and *QPRT* mRNA. (**C**) PCR was performed to detect miRNA-494-3p, miRNA-150-5p and miRNA-2467-3p expression after transfection with their inhibitors and mimics. (**D**) PCR was performed to detect *DCTPP1* or *QPRT* expression after transfection with miRNA-494-3p, miRNA-150-5p and miRNA-2467-3p inhibitors and mimics. (**E**) As per the RNA pull-down assay, both miRNA-150-5p and miRNA-2467-3p WT pulled down DSCAM-AS1 from the cell lysate (*P*<0.001, **E**), but their MT failed to do so. (**F**) The association between miRNA-150-5p and miRNA-2467-3p and the *QPRT* mRNA 3′ UTR was determined using a luciferase reporter assay. Transfection with miRNA-150-5p and miRNA-2467-3p mimics decreased the WT luciferase reporter activity; however, the transfection did not decrease the MT luciferase reporter activity. *P<0.05, **P<0.01 and ***P<0.001 vs. control group.

To determine whether the effect of DSCAM-AS1 on DCTPP1 and QPRT expression was due to miRNA competitive binding, we analyzed whether miRNA could bind to both DSCAM-AS1 and DCTPP1 or QPRT using PITA, RNA22, miRmap, mircoT, miRanda, PicTar, and Targetscan software. Two miRNAs could bind to both DSCAM-AS1 and DCTPP1, and eight miRNAs could bind to both DSCAM-AS1 and QPRT (Figure 5A). Among the two miRNAs, miRNA-494-3p was predicted to tightly bind to DSCAM-AS1; however, the connection between miRNA-494-3p and the *DCTPP1* mRNA seem to be weak due to fewer number of bases binding (Figure 5B). Two miRNAs among the eight miRNAs, miRNA-150-5p and miRNA-2467-3p, were predicted to tightly bind to both DSCAM-AS1 and the *QPRT* mRNA. To determine the regulatory effects of these miRNAs on DSCAM-AS1 and *QPRT* mRNA expression, we used the corresponding miRNA inhibitors and mimics to downregulate and upregulate the miRNAs, respectively. The change in miRNA-494-3p expression only conferred a moderate effect on *DCTPP1* mRNA levels in MCF-7 and T47D cells (Figure 5C and 5D). In contrast, downregulation of both miRNA-150-5p and miRNA-2467-3p significantly increased *QPRT* mRNA levels in MCF-7 and T47D cells (*P* < 0.05 or *P* < 0.01, Figure 5C and 5D); upregulation of these miRNAs showed the reverse effects (*P* < 0.05 or *P* < 0.01). As per the RNA pull-down assay, both miRNA-150-5p and miRNA-2467-3p wild type (WT) pulled down DSCAM-AS1 from the cell lysate (*P* < 0.001, Figure 5E), but their mutant type (MT) failed to do so. The association between miRNA-150-5p and miRNA-2467-3p and the *QPRT* mRNA 3′ UTR was determined using a luciferase reporter assay. Transfection with miRNA-150-5p and miRNA-2467-3p mimics decreased the WT luciferase reporter activity (*P* < 0.05 or *P* < 0.01, Figure 5F); however, the transfection did not decrease the MT luciferase reporter activity.

### DSCAM-AS1 regulated DCTPP1 expression by modulating histone acetylation

**Figure 6:**
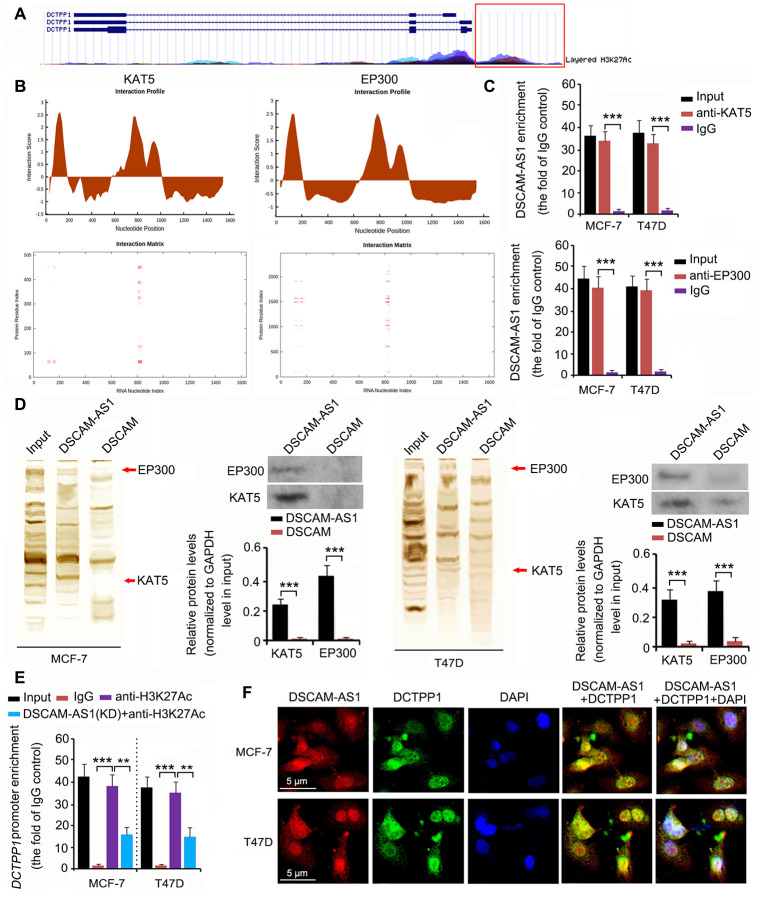
**DSCAM-AS1 regulated** DCTPP1 **transcription by modulating histone acetylation.** (**A**) The epigenetic regulation of DCTPP1 expression was analyzed using the UCSC Genome Browser Gateway. The DCTPP1 promoter was influenced by histone 3 acetylation at lysine 27. (**B**) As indicated by catRAPID, DSCAM-AS1 was predicted to bind to two key histone acetylases: KAT5 and EP300. The interaction matrix showed that bases 0 to 200 and 800 to 900 of DSCAM-AS1 probably have strong associations with both the KAT5 and EP300 proteins. (**C**) In the RIP assay, anti-KAT5, anti-EP300 antibodies, and IgG were used to separate KAT5-RNA, EP300-RNA, and IgG-RNA complexes from the cell lysate. DSCAM-AS1 was successfully detected in the KAT5-RNA and EP300-RNA complexes but not in the IgG-RNA complex. (**D**) In the RNA pull-down assay, bio-DSCAM and bio-DSCAM-AS1 were used to detect proteins that could bind to *DSCAM* mRNA and lncRNA DSCAM-AS1. Western blotting indicated that DSCAM-AS1, but not *DSCAM* mRNA, bound to KAT5 and EP300 proteins. (**E**) Results from ChIP assay showed that acetylated histone 3 bound to the DCTPP1 promoter. However, DSCAM-AS1 knockdown decreased acetylated histone 3 enrichment in the DCTPP1 promoter. (**F**) In FISH and immunofluorescence assays, both DSCAM-AS1 and DCTPP1 were observed in the cytoplasm and cell nucleus (**F**). **P<0.01 and ***P<0.001.

The epigenetic regulation of DCTPP1 expression was analyzed using the University of California Santa Cruz (UCSC) Genome Browser Gateway. The DCTPP1 promoter was influenced by histone 3 acetylation at lysine 27 (H3K27) (Figure 6A). Histone 3 acetylation is catalyzed by several histone acetylases. As indicated by catRAPID, DSCAM-AS1 was predicted to bind to two key histone acetylases: KAT5 and EP300 (Figure 6B). The interaction matrix showed that bases 0 to 200 and 800 to 900 of DSCAM-AS1 probably had strong associations with both the KAT5 and EP300 proteins. This hypothesis was tested using RNA immunoprecipitation (RIP) and RNA pull-down assays. In the RIP assay, anti-KAT5, anti-EP300 antibodies, and IgG were used to separate KAT5-RNA, EP300-RNA, and IgG-RNA complexes from cell lysate. DSCAM-AS1 was successfully detected in the KAT5-RNA and EP300-RNA complexes (all *P* < 0.001, Figure 6C), but not in the IgG-RNA complex. In the RNA pull-down assay, bio-DSCAM and bio-DSCAM-AS1 were used to detect proteins that could bind to *DSCAM* mRNA and the lncRNA DSCAM-AS1. Western blotting indicated that DSCAM-AS1, but not or very little *DSCAM* mRNA, bound to KAT5 and EP300 proteins (all *P* < 0.001, Figure 6D). We further performed a chromatin immunoprecipitation (ChIP) assay, which confirmed that acetylated histone 3 bound to the DCTPP1 promoter (all *P* < 0.001, Figure 6E). However, DSCAM-AS1 knockdown decreased acetylated histone 3 enrichment in the DCTPP1 promoter (*P* < 0.01). In the fluorescence in situ hybridization (FISH) and immunofluorescence assays, both DSCAM-AS1 and DCTPP1 were observed in the cytoplasm and cell nucleus (Figure 6F).

### DSCAM-AS1 protected *DCTPP1* mRNA from degradation mediated by miRNAs

**Figure 7:**
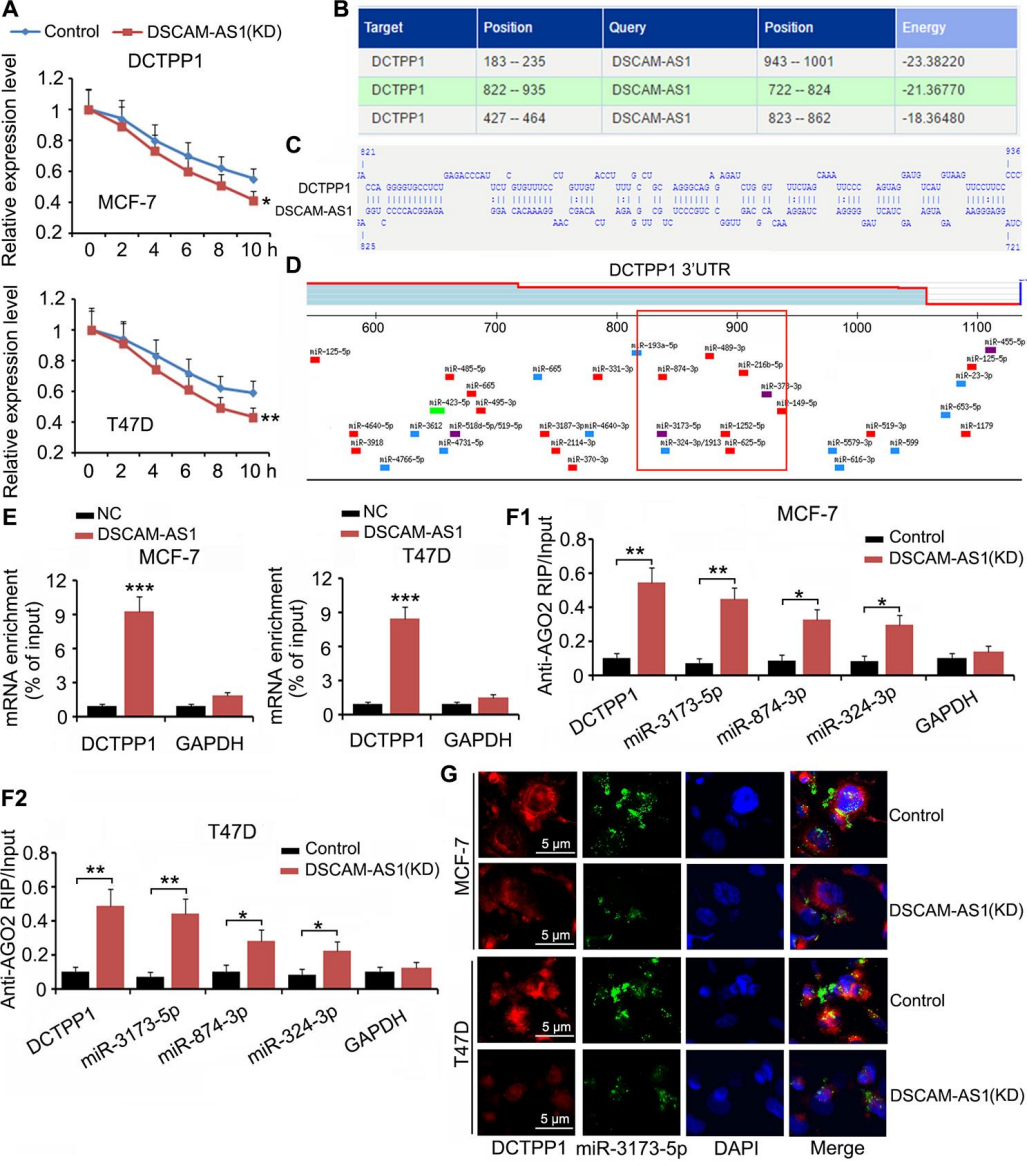
**DSCAM-AS1 protected** *DCTPP1* mRNA from degradation mediated by miRNAs. (**A**) In the mRNA stability assay, Actinomycin D was added to BC cells to prevent cell transcription. mRNA level was depicted at different time points, as measured by PCR. (**B**) Bioinformatics analysis using a RNA-RNA interaction software (http://rna.informatik.uni-freiburg.de/IntaRNA/Input.jsp) showed tight junctions between *DCTPP1* mRNA and DSCAM-AS1 at three areas. (**C**) There are more than 70 bases from 822 to 935 locus in *DCTPP1* mRNA bound to the bases in DSCAM-AS1. The region from 822 to 935 locus locates in the 3’UTR of *DCTPP1* mRNA. (**D**) Many miRNAs, such as miR-3173-5p, miR-874-3p and miR-874-3p, were predicted to bind to *DCTPP1* mRNA in the same region. (**E**) Interaction between *DCTPP1* mRNA and DSCAM-AS1 was confirmed by RNA-pull down test. (**F**) RIP analysis showed that DSCAM-AS1 knockdown increased the enrichment of *DCTPP1* mRNA, miR-3173-5p, miR-874-3p and miR-874-3p in the AGO2 protein complex when cell transcription was inhibited by Actinomycin D. (**G**) As indicated by FISH assay, DSCAM-AS1 knockdown decreased the levels of *DCTPP1* mRNA and miR-3173-5p in MCF-7 and T47D cells. *P<0.05 and **P<0.01.

Although we confirmed that DSCAM-AS1 promoted DCTPP1 transcription by modulating histone acetylation, an interesting phenomenon was that DCTPP1 expression was still regulated by DSCAM-AS1 when cell transcription was inhibited by Actinomycin D. In the mRNA stability assay, Actinomycin D was added to BC cells to prevent cell transcription; therefore, mRNA levels in cells decreased with time due to mRNA degradation. We found that DSCAM-AS1 knockdown facilitated the reduction of *DCTPP1* mRNA (*P* < 0.05 or *P* < 0.01, Figure 7A). Bioinformatics analysis using an RNA-RNA interaction software (http://rna.informatik.uni-freiburg.de/IntaRNA/Input.jsp) showed tight junctions between *DCTPP1* mRNA and DSCAM-AS1 at three areas (Figure 7B). For example, there were more than 70 bases (from 822 to 935) in the *DCTPP1* mRNA that were bound to DSCAM-AS1 bases (Figure 7C). The region from 822 to 935 was located in the 3ʹ UTR of *DCTPP1* mRNA. Many miRNAs, such as miR-3173-5p, miR-874-3p, and miR-874-3p, were predicted to bind to the *DCTPP1* mRNA in the same region (as shown in the red box, Figure 7D). Therefore, it was possible that the interaction between the *DCTPP1* mRNA and DSCAM-AS1 prevented these miRNAs binding to the *DCTPP1* 3′ UTR mRNA. Interaction between the *DCTPP1* mRNA and DSCAM-AS1 was confirmed by an RNA-pull down assay (Figure 7E). When cell transcription was inhibited by Actinomycin D, DSCAM-AS1 knockdown increased the enrichment of *DCTPP1* mRNA (*P* < 0.01), miR-3173-5p (*P* < 0.01), miR-874-3p (*P* < 0.05), and miR-874-3p (*P* < 0.05) in the argonaute RISC catalytic component 2 (AGO2) protein complex (Figure 7F). As indicated by the FISH assay, DSCAM-AS1 knockdown decreased the levels of *DCTPP1* mRNA and miR-3173-5p in MCF-7 and T47D cells (Figure 7G).

### Depletion of DSCAM-AS1 and knockdown of DCTPP1 and QPRT together affected BC cell growth and invasion

**Figure 8:**
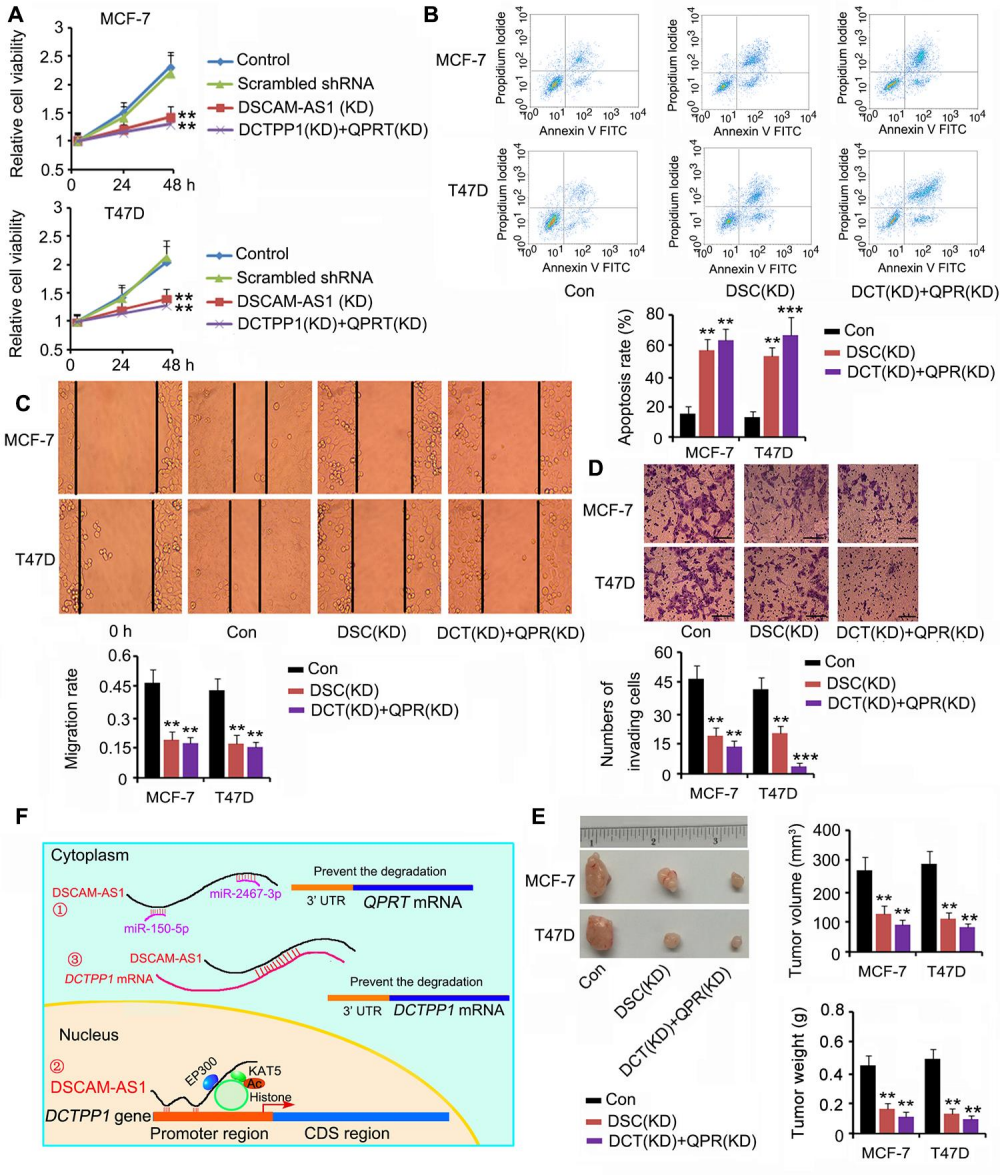
**Depletion of DSCAM-AS1 and knockdown of DCTPP1 and QPRT together affected BC cell growth and invasion.** Cell viability (**A**), apoptosis (**B**), migration (**C**), and invasion (**D**) assays were performed after silencing DSCAM-AS1 or DCTPP1 and QPRT together. DSCAM-AS1 depletion and knockdown of DCTPP1 and QPRT remarkably inhibited cell viability, migration, and invasion in MCF-7 and T47D cells; however, they promoted apoptosis. The bar in the pictures (**D**) indicates a length of 5 μm. (**E**) In the *in vivo* assay, silencing DSCAM-AS1 or DCTPP1 and QPRT together dramatically inhibited MCF-7 and T47D cell growth in nude mice. (**F**) The molecular mechanisms behind DSCAM-AS1 regulating DCTPP1 and QPRT. DSCAM-AS1 increased QPRT expression through sponging miRNA-150-5p and miRNA-2467-3p. In contrast, DSCAM-AS1 promoted the transcription of the *DCTPP1* gene by affecting H3K27 acetylation and enhancing *DCTPP1* mRNA stability through binding to the 3’UTR, which collectively resulted in the overexpression of DCTPP1. **P<0.01 and ***P<0.001 vs. control group. DSC(KD): DSCAM-AS1 knockdown; DCT(KD):DCTPP1 knockdown; QPR(KD): QPRT knockdown.

Based on our results, DSCAM-AS1 positively regulated both DCTPP1 and QPRT expression; thus, we silenced DSCAM-AS1 or DCTPP1 and QPRT together to determine their effects on BC cell growth and invasion. DSCAM-AS1 depletion and knockdown of DCTPP1 and QPRT remarkably inhibited cell viability, migration, and invasion (*P* < 0.01 or *P* < 0.001, Figure 8A, 8C and 8D); however, they promoted apoptosis (*P* < 0.01 or *P* < 0.001, Figure 8B). In the *in vivo* assay, silencing DSCAM-AS1 or DCTPP1 and QPRT together dramatically inhibited MCF-7 and T47D cell growth in nude mice (all *P* < 0.01, Figure 8E). Figure 8F shows the molecular mechanisms behind the regulation of DCTPP1 and QPRT by DSCAM-AS1, which are explained in the discussion section.

## DISCUSSION

We initially analyzed several databases to identify genes that were differentially expressed in BC and normal breast tissues. In combination with the analysis of gene expression and correlation of patient prognosis, we hypothesized that the *DCTPP1* and *QPRT* genes probably had an important role in BC development. Previous studies on DCTPP1’s effect on cancer are rare; however, these studies consistently suggest that DCTPP1 promotes the progression of some cancers [23–25]. Lu et al. found that autophagy induced by DCTPP1 overexpression is associated with poor clinical outcomes in prostate cancer [23]. Other studies suggest that DCTPP1 promotes 5-fluorouracil and decitabine resistance in stomach and cervical cancers [24, 25]. Song et al. show that DCTPP1 promotes the proliferation and stemness of BC MCF-7 and MDA-MB-231 cells, which are associated with DCTPP1-induced hypomethylation in cancer cells via intracellular 5-methyl-dCTP modulation [7]. In this study DCTPP1 overexpression was associated with suppressed apoptosis, enhanced migration, and enhanced invasion of BC MCF-7 and T47D cells while DCTPP1 deficiency promoted apoptosis and suppressed migration and invasion. These results suggest that DCTPP1 participated in various biological processes that promoted BC development.

The role of QPRT in BC has not been previously reported; however, its cancer-promoting effects have been observed in other cancers. A previous study noted the difference in QPRT expression between follicular thyroid adenoma (22%) and follicular thyroid carcinoma (65%) that includes both minimal invasive follicular carcinomas and widely invasive follicular carcinomas [26]. Oxidative stress, temozolomide, and irradiation were found to induce QPRT expression in malignant glioma cells. QPRT expression is associated with poor prognosis in recurrent glioblastomas after radiochemotherapy [10]. QPRT mediated a change in NAD(+) metabolism by exploiting microglia-derived quinolinic acid as an alternative source of replenishing intracellular NAD(+) pools when the classical *de novo* NAD(+) synthesis was blocked. Maintaining intracellular NAD(+) pools protects cancer cells from outside insults. Nevertheless, the imatinib resistance conferred by QPRT in leukemic cells is independent of NAD(+) [27]. We showed that QPRT was highly expressed in BC tissues compared to normal breast tissues. *In vitro* and *in vivo* studies further confirmed that QPRT promoted growth, migration, and invasion of BC cells but inhibited apoptosis. QPRT is thus a novel target for BC treatment.

Further, we demonstrated that DCTPP1 and QPRT expression in BC was associated with DSCAM-AS1 expression. Data in TCGA and GEO databases revealed that DCTPP1 and QPRT expression was positively correlated with DSCAM-AS1 expression, especially in ER-positive BC. Knockdown of DSCAM-AS1 in MCF-7 and T47D cells reduced DCTPP1 and QPRT expression, suggesting that they are positively regulated by DSCAM-AS1. As indicated by TCGA dataset, higher DSCAM-AS1 expression was associated with better prognosis in BC patients; however, overexpression of DSCAM-AS1 was conversely associated with poor clinical outcomes in both ER-positive and -negative BC. As BC can be divided into several types and subtypes, the role of DSCAM-AS1 in each type may be different, resulting in complex BC prognosis prediction. However, high expression of DSCAM-AS1 and many genes positively correlated with DSCAM-AS1 have been consistently associated with poor clinical outcomes, such as cancer aggression, tamoxifen resistance, higher grade, stage, and metastasis in ER-positive and luminal BC types [4, 28].

A crucial role of lncRNAs in the cytoplasm is that they function as ceRNAs of miRNAs, preventing miRNA-mediated mRNA degradation. DSCAM-AS1 has been shown to competitively bind miR-137 and increase epidermal growth factor receptor pathway substrate 8 expression in tamoxifen-resistant BC [29]. As both *DCTPP1* and *QPRT* mRNA levels were positively regulated by DSCAM-AS1, we investigated whether the regulatory effect of DSCAM-AS1 was dependent on miRNAs. The results revealed that DSCAM-AS1 positively regulated *QPRT* mRNA levels by competitively binding both miRNA-150-5p and miRNA-2467-3p. In contrast, miRNA-494-3p was predicted to weakly bind to *DCTPP1* mRNA; thus, knockdown of miRNA-494-3p only moderately increased *DCTPP1* mRNA levels. Neither miRNA-150-5p nor miRNA-2467-3p has been reported in BC. However, miR-150-5p has been found to be significantly downregulated in both cancer tissue and plasma of patients with prostate cancer [30]. Additionally, lncSNHG14 promotes the development and progression of bladder cancer by competitively binding miR-150-5p [31].

Many lncRNAs in the nucleus have been implicated in epigenetic gene regulation. For example, lncRNA NEAT1 regulates H3K27 acetylation and crotonylation near the transcription start sites of many endocytosis-related genes, affecting the expression of these genes [32]. We showed that *DCTPP1* gene expression was also influenced by H3K27 acetylation. Moreover, DSCAM-AS1 was predicted to bind the histone acetylases KAT5 and EP300. Therefore, DSCAM-AS1 may function as a “scaffold” on the *DCTPP1* gene promoter to recruit KAT5 and EP300 and influence H3K27 acetylation. The electrostatic interaction of the positive charges on histones and the negative charges on DNA leads to a condensed and repressive chromatin structure. However, H3K27 acetylation attenuates positive charges, reducing the affinity of histones for negatively-charged DNA, resulting in a relaxed chromatin structure that is amenable to transcription initiation. RIP and RNA pull-down assays consequently confirmed the association of DSCAM-AS1 with KAT5 and EP300. The connection between DSCAM-AS1 and EP300 has also been validated by Miano et al [33]. In the ChIP assay, the enrichment of acetylated histone 3 at K27 in the *DCTPP1* gene promoter was decreased after knocking down DSCAM-AS1, suggesting that DSCAM-AS1 promoted H3K27 acetylation in the gene promoter.

Although we confirmed that DSCAM-AS1 promoted DCTPP1 transcription by modulating histone acetylation, an interesting phenomenon was that DCTPP1 expression was still influenced by DSCAM-AS1 when cell transcription was inhibited by Actinomycin D. This result suggested that there were other molecular mechanisms underlying the regulatory effect of DSCAM-AS1 on DCTPP1 expression. Bioinformatics analysis revealed a tight junction between DSCAM-AS1 and *DCTPP1* mRNA at the 3′ UTR. Since the 3′ UTR was also the primary region targeted by miRNAs, the combination between DSCAM-AS1 and *DCTPP1* mRNA probably prevented miRNA-targeting to the mRNA. A similar mechanism was also observed in other studies. Faghihi found that a lncRNA BACE1-AS forms an RNA duplex with *BACE1* mRNA, which increases the stability and expression of *BACE1* mRNA [34]. In addition, lncRNA PXN-AS1-L increases the stability and expression of *SAPCD2* mRNA through binding to the 3′ UTR; increased SAPCD2 promotes the malignancy of nasopharyngeal carcinoma [35]. The function of lncRNAs to increase mRNA stability is different from that of lncRNAs competitively binding to miRNAs, which prevents miRNA-targeting to the mRNA, though both lncRNA functions disrupt mRNA degradation mediated by miRNAs. This study initially confirmed the combination between DSCAM-AS1 and *DCTPP1* mRNA. When cell transcription was inhibited by Actinomycin D, the knockdown of DSCAM-AS1 increased the enrichment of *DCTPP1* mRNA levels and some miRNAs in the AGO2 protein complex, but decreased the total *DCTPP1* mRNA levels in cells. These data suggested that DSCAM-AS1 increased *DCTPP1* mRNA stability via the formation of an RNA duplex.

As DSCAM-AS1 positively regulated both DCTPP1 and QPRT, the DSCAM-AS1 deficiency probably conferred a cancer-inhibitory effect, such as knocking down both DCTPP1 and QPRT. Indeed, DSCAM-AS1 deficiency remarkably inhibited BC cell growth, migration, and invasion, and promoted apoptosis. The limitation of this study was that, although we confirmed the roles of DCTPP1 and QPRT in promoting the proliferation and invasion in ER-positive BC, the underlying mechanism was not fully understood. As has been extensively reported, both DCTPP1 and QPRT play important roles in maintaining DNA integrity [6–10]. DNA integrity is critical for cell proliferation, and even cell survival under the challenge of radiation and DNA-targeting drugs. Therefore, the effects of DCTPP1 and QPRT on DNA function are probably associated with their cancer-promoting effects. Moreover, these effects could render cancer resistance during radiotherapy and chemotherapy, which should be a focus of future studies.

In summary, this study showed that overexpression of DCTPP1 and QPRT promoted the growth, migration, and invasion of ER-positive BC. DSCAM-AS1 was responsible for the upregulation of DCTPP1 and QPRT in BC. As shown in Figure 8F, DSCAM-AS1 increases QPRT expression through competitive binding of miRNA-150-5p and miRNA-2467-3p. In contrast, DSCAM-AS1 promoted the transcription of the *DCTPP1* gene by affecting H3K27 acetylation and enhanced *DCTPP1* mRNA stability via binding to the 3′ UTR, which collectively resulted in the overexpression of DCTPP1. Since both DCTPP1 and QPRT were positively regulated by DSCAM-AS1, its knockdown inhibited the growth, migration, and invasion of ER-positive BC.

## MATERIALS AND METHODS

### Bioinformatics analysis

Microarray datasets, including GSE42568 [BC tissues (n = 104); adjacent normal tissues (n = 17)], GSE65194 [BC tissues (n = 153); adjacent normal tissues (n = 11)], and TCGA [BC tissues (n = 1109); adjacent normal tissues (n = 113)] were selected for bioinformatics analysis. Venn diagrams were used to identify genes differentially expressed between BC and adjacent normal tissues in the GEO and TCGA datasets. Potential miRNAs targeting DCTPP1, QPRT, and DSCAM-AS1 were detected using the PITA, RNA22, miRmap, mircoT, miRanda, PicTar, and Targetscan software (https://web.archive.org/web/20110201054358/http://starbase.sysu.edu.cn/clipSeq.php). Epigenetic regulation of DCTPP1 expression was analyzed using the UCSC Genome Browser Gateway (http://www.genome.ucsc.edu/cgi-bin/hgGateway?redirect=manual&source=www.genome.ucsc.edu). The interaction between DSCAM-AS1 and histone acetylases was evaluated using catRAPID (http://service.tartaglialab.com/page/catrapid_group) and RPISeq (RNA-protein interaction prediction, http://pridb.gdcb.iastate.edu/RPISeq/).

### Collection of BC tissue samples

Tumor samples were collected from female patients with BC (age range: 30–65 years) undergoing breast mass resection at Harbin Medical University Cancer Hospital (Harbin, China) between July 2017 and July 2018. The research protocol was approved by the Ethics Committee of Harbin Medical University, and all patients involved provided written informed consent. BC (n = 27) and adjacent normal tissues (n = 27) were included in the study. Tissues samples were collected before chemotherapy. Patient clinical data are presented in Table 1.

**Table 1:** Clinicopathological characteristics of patients with breast cancer.

	**Case (n)**
Age, years	
<50	15
≥50	12
Tumor size, cm	
<2	10
≥2	17
Lymph node infiltrated	
No	17
Yes	10
TNM stage	
Stage I/II	18
Stage III/IV	11
Pathological grade	
G1	7
G2	11
G3	9
ER status	
Negative	5
Positive	22
PR status	
Negative	9
Positive	18
HER2 status	
Negative	10
Positive	17

TNM: Tumor-Node-Metastasis; ER: estrogen receptor; PR: progesterone receptor; HER2: receptor tyrosine-protein kinase erbB-2.

### Immunocytochemistry analysis

Tissues were fixed in 10% formalin. Paraffin-embedded sections from the tissue specimens were deparaffinized and heated at 97 °C in 10 mM citrate buffer (pH 6.0) for 20 min to retrieve antigens. Tissue sections were incubated with anti-DCTPP1 (ab224051, Abcam, Cambridge, MA, USA) and anti-QPRT (ab171944, Abcam) overnight at 4 °C followed by horseradish peroxidase (HRP)-labeled anti-rabbit IgG (1:200; Abcam) at 37 °C for 30 min. Each section was immersed in 500 μL of a diaminobenzidine working solution at room temperature for 3–10 min.

### Cell lines and culture conditions

DSCAM-AS1 is highly expressed in ER-positive BC cells because *DSCAM-AS1* is pre-transcriptionally regulated by the ER [4]. This study selected two ER-positive BC cell lines, MCF-7 and T47D, purchased from the American Type Culture Collection (Manassas, VA, US). MCF7 cells were cultured in Dulbecco’s modified Eagle’s medium (DMEM, Invitrogen, Thermo Fisher Scientific, Inc. Shanghai, China) containing 10% fetal bovine serum (FBS; Hyclone, GE Healthcare Life Sciences) and 1% penicillin-streptomycin. T47D cells were maintained in Roswell Park Memorial Institute (RPMI, Invitrogen) 1640 medium supplemented with 10% FBS and 1% penicillin-streptomycin. The cell lines were routinely maintained at 37 °C in a 5% CO_2_ atmosphere.

### Transfection

shRNAs against DCTPP1, QPRT, and DSCAM-AS1 were obtained from GenePharma Co., Ltd. (Shanghai, China). shRNAs sequences are similar to those reported in previous studies [4, 7, 10]. Lentiviral constructs (pGLVU6/GFP) containing shRNAs or scrambled shRNAs were transfected into MCF-7 and T47D cells using the Lipofectamine 2000 kit (Invitrogen) after cell growth to 60–80% confluence. To overexpress DCTPP1 and QPRT, their predominant isoforms were cloned into the enhanced green fluorescent protein plasmid-C1 vector (GenePharma) to construct an overexpression vector containing DCTPP1 and QPRT. Transfection of MCF-7 and T47D cells with the vectors and control vectors was achieved using Lipofectamine 2000. Cells were collected 48 h after transfection.

Inhibitors of miRNA-494-3p (5′-GAGGUUUCCCGUGUAUGUUUCA-3′), miRNA-150-5p (5′-CACUGGUACAAGGGUUGGGAGA-3′), and miRNA-2467-3p (5′-CCUGAGCCUCUCUGCCUCUGCU-3′) and their mimics were constructed by the GenePharma company and transfected into MCF-7 and T47D cells using Lipofectamine 2000 (Invitrogen) following standard protocols. Cells were harvested 24 h after transfection for further experiments.

### 3-4,5-dimethylthia-zolyl-2)-2,5-diphenyltetrazolium bromide (MTT) assay

In brief, 1×10^4^ cells/well were plated in 96-well plates and cultured to 80% confluence. After cells were exposed to the indicated treatments, cells were washed twice with phosphate buffered saline (PBS) and further incubated with 0.5 mg/mL MTT for 2 h. The blue formazan crystals were solubilized with dimethyl sulfoxide (DMSO, 100 μL/well), and the absorbance of blue-dyed solution was read at a wavelength of 550 nm. Cell viability was used to compare the absorbance to untreated control cells.

### Apoptosis assay

Apoptosis was evaluated using the Annexin V-fluorescein isothiocyanate (FITC)/Propidium Iodide (PI) Staining Kit (Dojindo Molecular Technologies, Inc., Dalian, China). After the indicated treatments, cells were harvested and resuspended in PBS at a concentration of 1×10^6^ cells/mL. A 100 μL sample of the cell suspension was mixed with 5 μL Annexin V-FITC and 5 μL PI. The mixture was incubated for 15 min at room temperature in the dark and analyzed using a FACS Calibur Flow Cytometer (Beckman Coulter, CytoFLEX S, United States).

### Wound healing assay

MCF-7 and T47D cells were cultured in 6-well plates until confluent. Following the indicated treatments, a 1 mL pipette tip was used to create a “scratch-wound” on the cell monolayer. The culture medium was replaced with FBS-free medium. Microscopic images of the cells were captured immediately after scratching and 24 h after scratching. The cell migration rate was calculated based on the movement of cells from initial placement to the final distance travelled after 24 h.

### Invasion assay

Transwell plates (Corning, Shanghai, China) containing polycarbonate filters with a pore size of 8.0 μm were used in invasion assays to evaluate cell invasion capacity. The transwell filters were first coated with 50 μL of 1 mg/mL Matrigel® matrix (Becton Dickinson, Franklin Lakes, NJ, USA) at 37 °C for 4 h to allow gelling. MCF-7 and T47D cells were seeded at a density of 1.5×10^5^ cells in 200 μL of medium without FBS in the upper chamber. FBS-containing culture medium was added to the lower chamber. The filters were removed and washed in PBS. The cells in the upper surface of the filters were removed with a cotton bud. The inserts were fixed in cold methanol for 10 min at room temperature and stained with hematoxylin. Cells on the lower surface were counted in 10 fields at 200x magnification.

### Quantitative polymerase chain reaction (qPCR)

Total cellular RNA extraction was performed using TRIzol® reagent (Thermo Fisher Scientific, Inc.). RNA was reverse transcribed into cDNA for 1 h at 37 °C and 5 min at 85 °C using SuperScript II reverse transcriptase with RNase H (Invitrogen, Grand Island, NY, USA). qPCR was performed using IQ SYBR Green Supermix (Invitrogen). The thermocycling conditions were set as follows: initial denaturation at 95 °C for 60 s, denaturing at 95 °C for 20 s, annealing at 58 °C for 30 s, and extension at 72 °C for 30 s, for 43 cycles, followed by a melting curve from 65–95 °C with increments of 0.5 °C for 5 s in an iCycleriQTX detection system (Bio-Rad Laboratories, Inc.). All results were normalized to GAPDH, and the 2^-ΔΔCq^ method was used to quantify the fold change. The primer information was shown in Table 2

**Table 2:** The primer sequences used in qPCR.

**Gene name**	**Primer orientation**	**Sequences**
DCTPP1	Forward	5'-CGCCTCCATGCTGAGTTTG-3'
	Reverse	5'-CCAGGTTCCCCATCGGTTTTC-3'
QPRT	Forward	5'-GGGCAGCCTTTCTTCGATG-3'
	Reverse	5'-GGAGCCCATACTTCTCCACCA-3'
DSCAM-AS1	Forward	5'-GTGACACAGCAAGACTCCCT-3'
	Reverse	5'-GATCCGTCGTCCATCTCTGT-3'
miR-494-3p	Forward	5'-ACACTCCAGCTGGGTGAAACATACACGGGA-3'
	Reverse	5'-CTCAACTGGTGTCGTGGAGTCGGCAATTCAGTTGAGGAGGTTTC-3'
miR-150-5p	Forward	5'-ACACTCCAGCTGGGTCTCCCAACCCTTGTA-3'
	Reverse	5'-CTCAACTGGTGTCGTGGAGTCGGCAATTCAGTTGAGCACTGGTA-3'
miR-2467-3p	Forward	5'-ACACTCCAGCTGGGAGCAGAGGCAGAGAGG-3'
	Reverse	5'-CTCAACTGGTGTCGTGGAGTCGGCAATTCAGTTGAGCCTGAGCC-3'
miR-3173-5p	Forward	5'-ACACTCCAGCTGGGTGCCCTGCCTGTTTTC-3'
	Reverse	5'-CTCAACTGGTGTCGTGGAGTCGGCAATTCAGTTGAGAAAGGAGA-3'
miR-874-3p	Forward	5'-ACACTCCAGCTGGGCTGCCCTGGCCCGAGG-3'
	Reverse	5'-CTCAACTGGTGTCGTGGAGTCGGCAATTCAGTTGAGTCGGTCCC-3'
miR-324-3p	Forward	5'-ACACTCCAGCTGGGACTGCCCCAGGTGC-3'
	Reverse	5'-CTCAACTGGTGTCGTGGAGTCGGCAATTCAGTTGAGACTGCCCC-3'
GAPDH	Forward	5'-GGGTGTGAACCATGAGAAGT-3'
	Reverse	5'-TGAGTCCTTCCACGATACCAA-3'
U6	Forward	5'-CTCGCTTCGGCAGCACA-3'
	Reverse	5'-AACGCTTCACGAATTTGCGT-3'

### Western blot assay

Western immunoblot assays were performed by separating cell lysates on 4–12% sodium dodecyl sulfate (SDS) polyacrylamide gels (Novex) to separate proteins. The proteins were transferred to polyvinylidene difluoride (PVDF) membranes (Pierce; Thermo Fisher Scientific, Inc.), and the membranes were blocked with 5% skim milk in Tris-buffered saline containing 0.05% Tween-20 (TBST). Membranes were subsequently incubated overnight at 4 °C with anti-DCTPP1 (ab224051, Abcam), anti-QPRT (ab171944, Abcam), anti-KAT5 (ab151432, Abcam), anti-EP300 (ab14984, Abcam), and anti-GAPDH antibodies (ab181602, Abcam), followed by incubation with horseradish peroxidase-conjugated secondary antibodies at room temperature for 2 h. Protein bands were revealed using chemiluminescence according to procedures given by the enhanced chemiluminescence (ECL) detection kit (Pierce, Thermo Scientific, Rockford, IL, USA).

### Dual luciferase reporter assay

The 3′ UTR of QPRT was amplified and cloned into the pmirGLO vector (XhoI and NotI restriction enzyme sites; Promega, Madison, WI, USA) to construct the QPRT WT reporter. The putative target regions of miRNA-150-5p and miRNA-2467-3p on the QPRT 3′ UTR sequence were mutated and cloned into the pmirGLO vector to construct QPRT MT reporters. The MCF-7 and T47D cells were seeded onto 12-well plates with a density of 1×10^4^ cells/well and transfected with the WT or MT reporters together with either miRNA-150-5p mimics (GenePharma), miRNA-2467-3p mimics (GenePharma) or NC using Lipofectamine 2000 (Invitrogen). Cells were harvested at 48 h and the activity of firefly luciferase was normalized to that of renilla luciferase.

### RNA immunoprecipitation assays

RIP assays were performed using a Millipore EZ-Magna RIP RNA-Binding Protein Immunoprecipitation kit (Millipore, #17-700) according to the manufacturer’s instructions. Briefly, cell pellets were lysed in radio immunoprecipitation assay buffer containing RNase inhibitor and protease inhibitor cocktail, followed by sonication on ice and subsequent DNase treatment for 30 min. Immunoprecipitations were performed using protein A/G agarose beads with anti-KAT5, anti-EP300, and anti-AGO2 antibodies or the equivalent IgG. The RIP RNA product was detected by qRT-PCR, as described above.

### RNA pull-down assay

To confirm the association between miRNA-150-5p and miRNA-2467-3p and DSCAM-AS1, biotinylated RNA probes including Bio-miR-NC, Bio-miRNA-150-5p WT, Bio-miRNA-150-5p MT, Bio-miRNA-2467-3p WT, and Bio-miRNA-2467-3p MT were synthesized by KeyGen Biotech Company (Shanghai, China). The RNA probes were incubated with BC cells lysates and extracted using streptavidin-coupled magnetic beads according to the instructions from the Pierce™ Magnetic RNA Pull-Down Kit (Rockford, IL, USA). RNA-RNA complexes were eluted using salt solution and purified using TRIzol® (Pierce). qPCR was performed to detect the enrichment of DSCAM-AS1 in the RNA-RNA complexes.

To investigate the interaction between DSCAM-AS1 and two histone acetylases, KAT5 and EP300, DSCAM-AS1 (sense) and DSCAM (anti-sense) were transcribed *in vitro* and biotin-labeled using T7 RNA polymerase (Roche) and Biotin RNA Labeling Mix (Roche). After treatment with DNase I (Takara) to remove DNA and purification using the RNeasy Mini Kit (Qiagen, Shenzhen, China), 1 μg of whole-cell lysate was incubated with 3 mg of biotinylated DSCAM-AS1 (Bio-DSCAM-AS1) and DSCAM (Bio-DSCAM) overnight at 4 °C. RNA and protein complexes were isolated with streptavidin agarose beads (Invitrogen, USA). Eluates were analyzed by mass spectrometry. The KAT5 and EP300 proteins in the RNA-protein complex were detected using western blotting, as described above.

Bio-DSCAM-AS1 was also used to analyze the interaction between DSCAM-AS1 and *DCTPP1* mRNA. Bio-DSCAM-AS1 was incubated with the BC cell lysates and extracted using streptavidin-coupled magnetic beads according to the instructions from the Pierce™ Magnetic RNA Pull-Down Kit (Rockford). RNA-RNA complexes were eluted using the salt solution and purified using TRIzol® (Pierce). qPCR was performed to detect the enrichment of *DCTPP1* mRNA in the RNA-RNA complexes.

### Chromatin immunoprecipitation assay

Cells were fixed using 1% formaldehyde and harvested on ice with ChIP lysis buffer (50 mM Tris-HCl pH 8.0, 5 mM ethylenediaminetetraacetic acid (EDTA), 0.1% deoxycholate, 1% Triton X-100, 150 mM NaCl, and proteinase inhibitors). Total extracted chromatin was sonicated to an average size of 250–500 bp using an immersion sonicator. Chromatin extracts were diluted with immunoprecipitation (IP) buffer (1% Triton X-100, 2 mM EDTA, 20 mM Tris-HCl of pH 8.1, 150 mM NaCl, supplemented with complete protease inhibitors) and incubated overnight with the anti-histone H3 (acetyl K27) antibody (ab203953, Abcam) or IgG at 4 °C on a rotating platform. Protein A and G sepharose-beads (GE Healthcare Life Sciences) were pre-coated with IP buffer supplemented with 5% bovine serum albumin to reduce nonspecific antibody binding. After incubation of protein A or G sepharose beads for 2 h, beads were washed sequentially for 5 min on a rotating platform. Finally, the DNA fraction in the *DCTPP1* gene promoter was analyzed using qPCR. DNA enrichment was normalized to input samples (1% of total chromatin used per IP) and expressed as fold enrichment of specific binding over the control nonspecific IgG binding.

### RNA FISH and immunofluorescence

The DSCAM-AS1 RNA FISH assay was combined with immunofluorescence detection of DCTPP1 protein to understand their colocalization. Moreover, another RNA FISH assay was performed to analyze the interaction between *DCTPP1* mRNA and miR-3173-5p. The oligonucleotides (probes) targeted to DSCAM-AS1, *DCTPP1* mRNA, and miR-3173-5p were designed by Biosearch Technologies (Shanghai, China). BC cells were fixed in 3.7% formaldehyde in PBS (pH 7.4) for 10 min at room temperature and permeabilized in 70% ethyl alcohol at 4 °C at least 1 h. Probes were hybridized to fixed cells according to the manufacturer’s protocol (Biosearch Technologies). Afterwards, anti-DCTPP1 primary antibody was incubated for 2 h at 37 °C. 4′,6-diamidino-2-phenylindole (DAPI) was finally added to cells to stain the nucleus. Images were acquired using a laser confocal microscope (Leica SP5, Heidelberg, Germany).

### mRNA stability assay

Actinomycin D was added at a concentration of 5 mg/mL to inhibit intracellular RNA synthesis. At stipulated time points, total RNA from the cells was obtained using the TRIzol® reagent (Invitrogen). RNA quantities were determined using qPCR.

### Tumor xenografts in nude mice

The animal experiment was approved by the Ethical Committee for Animal Research of Harbin Medical University (protocol number: 2017-020). BALB/c nude mice (4–5 weeks old males) were purchased from the Central Animal Facility of Harbin Medical University. Mice were housed in plastic cages with sealed air filters (28 °C; 40–60% humidity; 10 h light and 14 h dark per day). Six mice were randomly allocated to each group. MCF-7 and T47D cells (200 mL, 1×10^6^) were injected into the left side of each mouse’s back. The mice were sacrificed through cervical vertebra dislocation 16 days after the cell injection. The tumor volume was calculated using the equation v = a×b^2^/2.

### Statistical analyses

All experiments were performed at least three times. Experimental data were examined using either a one-way analysis of variance or an unpaired two-tailed *t*-test with GraphPad Prism software (GraphPad Software, San Diego, CA, USA). A value of *P* < 0.05 was set as the statistical significance level.
