# Correction for: Silencing DSCAM-AS1 suppresses the growth and invasion of ER-positive breast cancer cells by downregulating both DCTPP1 and QPRT

**DOI:** 10.18632/aging.203837

**Published:** 2022-01-15

**Authors:** Zhang Yue, Jia Shusheng, Song Hongtao, Zhao Shu, Huang Lan, Zhang Qingyuan, Cheng Shaoqiang, Huang Yuanxi

**Affiliations:** 1 Department of Medical Oncology, Harbin Medical University Cancer Hospital, Harbin, China; 2 Department of Breast Surgery, Harbin Medical University Cancer Hospital, Harbin, China; 3 Department of Pathology, Harbin Medical University Cancer Hospital, Harbin, China

Original article: Aging. 2020; 12:14754–14774. 14754-14774 . https://doi.org/10.18632/aging.103538

**This article has been corrected:** The authors recently found an error in **Figure 2D**. The flow cytometry dot plot for the shRNA-QPRT group of T47D cells in **Figure 2D** was mixed up with the dot plot for the T47D DSC(KD) group in **Figure 8B**. The authors corrected panel **2D** in **Figure 2** by using a representative image of the T47D shRNA-QPRT group from the original set of experiments. This alteration does not affect the results or conclusions of this work. The authors would like to apologize for any inconvenience caused.

New **Figure 2** is presented below.

**Figure 2:**
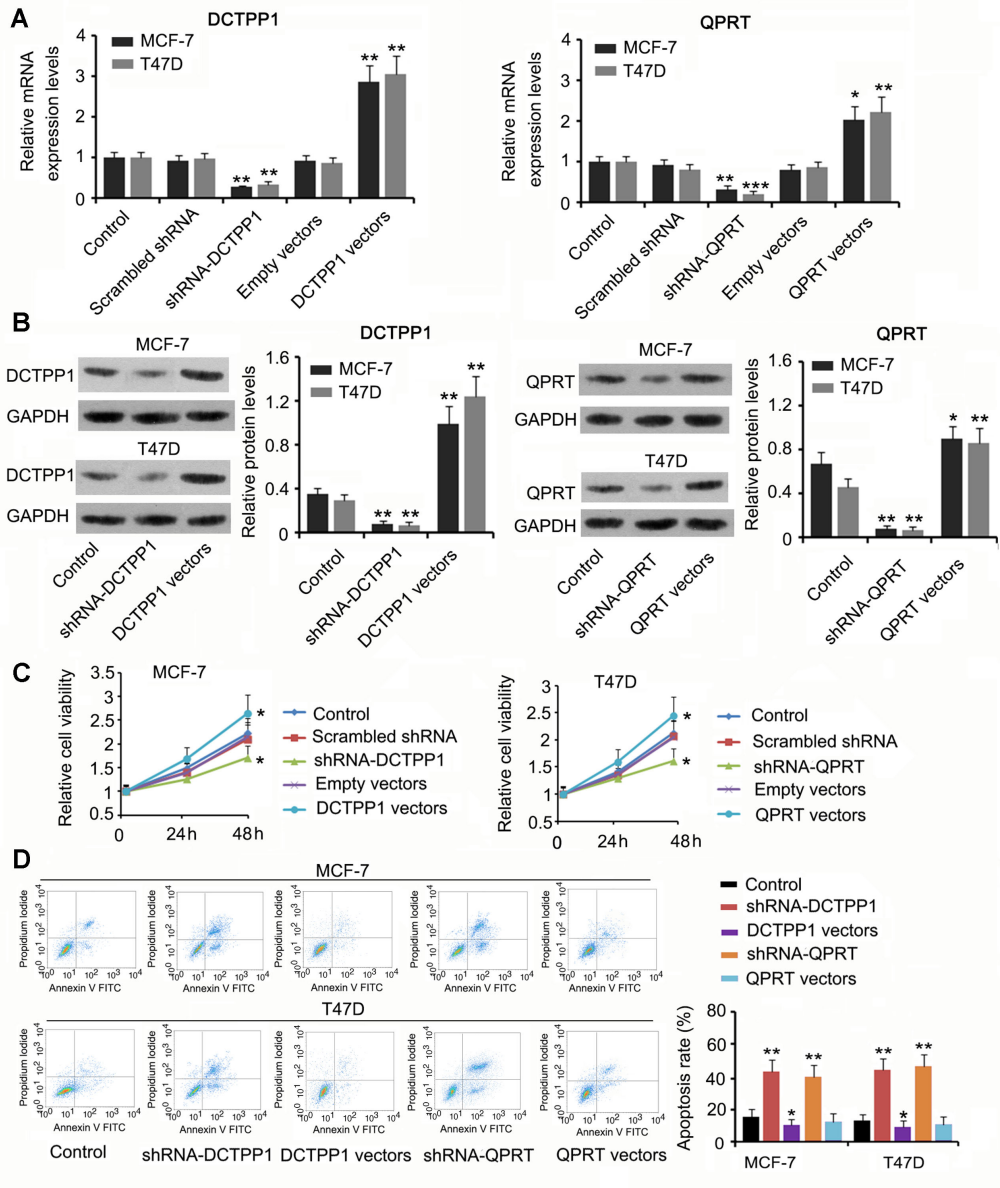
**The regulatory effects of DCTPP1 and QPRT on BC cell growth and apoptosis. **DCTPP1 and QPRT mRNA (**A**) and protein (**B**) levels in MCF-7 and T47D cells were changed after transfection with shRNA-DCTPP1, shRNA-QPRT, and DCTPP1 and QPRT expression vectors. (**C**) Down-regulation of DCTPP1 or QPRT was associated with reduced viability in both MCF-7 and T47D cells and increased DCTPP1 or QPRT was associated with increased cell viability. (**D**) The apoptosis rate of MCF-7 and T47D cells increased after knocking down either DCTPP1 or QPRT. DCTPP1 overexpression decreased the apoptosis rate of MCF-7 and T47D cells, and QPRT overexpression only marginally decreased MCF-7 and T47D apoptosis. *P<0.05, **P<0.01 and ***P<0.001 vs. control group.

